# Electrical Double Layer Capacitance in a Graphene-embedded Al_2_O_3_ Gate Dielectric

**DOI:** 10.1038/srep16001

**Published:** 2015-11-04

**Authors:** Bok Ki Min, Seong K. Kim, Seong Jun Kim, Sung Ho Kim, Min-A Kang, Chong-Yun Park, Wooseok Song, Sung Myung, Jongsun Lim, Ki-Seok An

**Affiliations:** 1 Thin Film Materials Research Center, Korea Research Institute of Chemical Technology (KRICT), Yuseong P. O. Box 107, Daejeon 305-600, Republic of Korea; 2 Department of Physics, Sungkyunkwan University, 2066 Seobu-ro, Jangan-gu, Suwon-si, Gyeonggi-do 440-746, Republic of Korea; 3 Department of Chemical Engineering and Materials Science, University of California, Davis, California 95616-5294, USA

## Abstract

Graphene heterostructures are of considerable interest as a new class of electronic devices with exceptional performance in a broad range of applications has been realized. Here, we propose a graphene-embedded Al_2_O_3_ gate dielectric with a relatively high dielectric constant of 15.5, which is about 2 times that of Al_2_O_3_, having a low leakage current with insertion of tri-layer graphene. In this system, the enhanced capacitance of the hybrid structure can be understood by the formation of a space charge layer at the graphene/Al_2_O_3_ interface. The electrical properties of the interface can be further explained by the electrical double layer (EDL) model dominated by the diffuse layer.

The fabrication of field effect transistors (FETs) with low operating voltage and input power has been researched for decades. According to the drain current equation of FETs, reduction of operating voltage and input power can be achieved with enhancement of the mobility of channel materials or the capacitance of the gate dielectric^1^. In order to increase the capacitance of the gate dielectric, processing of an ultra-thin SiO_2_ gate dielectric layer of a few nanometers was realized. However, the leakage current due to the quantum tunneling effect has become a significant problem, which limits further scale down of the SiO_2_ layer. Alternatively, the use of high-k dielectric materials such as hafnium oxide (HfO_2_), zirconium oxide (ZrO_2_), and aluminum oxide (Al_2_O_3_) that may provide high capacitance without scaling down to nano-sizes has received a great deal of interest^2^,^3^,^4^,^5^,^6^,^7^,^8^. Beside these metal oxides, alternative gate dielectrics with a high dielectric constant are also being considered, such as organic/inorganic nanocomposites^9^,^10^, ionic gels^11^,^12^,^13^,^14^, metal ion incorporated alumina^15^,^16^, and conductive carbon/polymer composites^17^,^18^,^19^,^20^. In these cases, the mechanism of the high capacitance has been explained via ionic polarization and interfacial polarization due to space charge.

Meanwhile, graphene, which is well known for its unique properties due to carbon-based two-dimensional structures, has a relatively low density of states compared to other metals because of its linear energy-momentum relationship near the Dirac point^21^. Thus, when graphene is in contact with other materials, the interface strongly affects the properties of graphene and the performance of the overall device^22^,^23^,^24^,^25^. Polymer dielectrics implanted with graphene also have been studied before and have shown capacitance enhancement of few factors to over an order of magnitude as reported by Ruoff *et al.*^26^, but the detailed mechanism for the enhancement of capacitance remains elusive.

Here, we propose thermal chemical vapor deposition (TCVD)-grown graphene-embedded Al_2_O_3_ capacitors deposited by atomic layer deposition (ALD) with a relatively high dielectric constant and negligible dielectric loss. For the characterization of the interface between graphene and Al_2_O_3_, we measured the frequency and gate voltage dependence of capacitance by varying the thickness of the Al_2_O_3_ layer and the number of graphene layers for further analysis of the space charge layer. With the obtained results, we also suggest a reason for enhanced capacitance, which is the formation of an electrical double layer (EDL) due to the space charge at the interface.

## Results

### Preparation and characterization of a graphene-embedded Al_2_O_3_ capacitor

**Figure 1:**
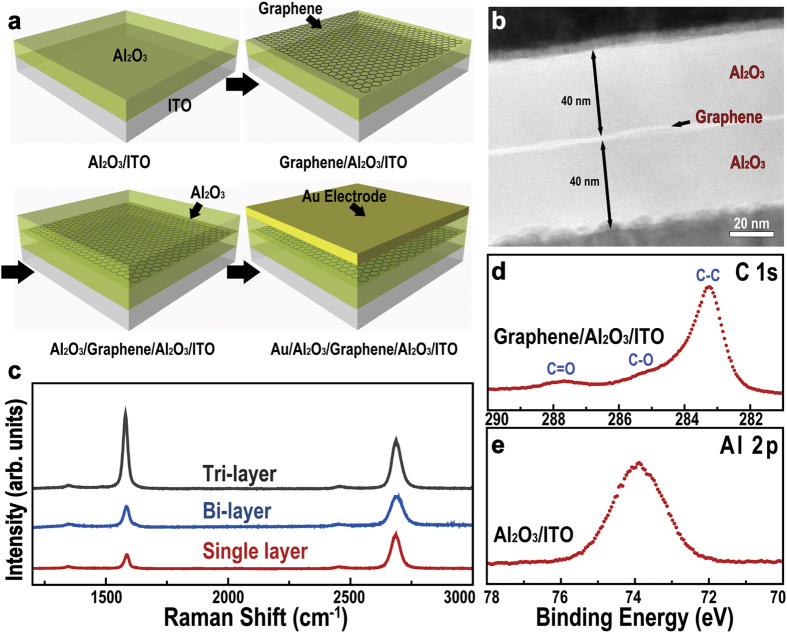
Preparation and characterization of a graphene-embedded Al_2_O_3_ capacitor. (**a**) Schematic of the graphene-embedded Al_2_O_3_ capacitor fabrication process. (**b**) TEM cross-sectional image of the graphene-embedded Al_2_O_3_ capacitor. (**c**) Raman spectrum of graphene sheets as a function of the number of layers (single, bi-, and tri-layer). XPS spectra of (**d**) C 1s for graphene on Al_2_O_3_ and (**e**) Al 2p for Al_2_O_3_ layer on ITO.

Figure 1a shows the fabrication process of a graphene-embedded metal-insulator-metal (MIM) capacitor. Al_2_O_3_ films were deposited on cleaned ITO-coated glass using the ALD process and the graphene was synthesized utilizing the same method as previous works (see Methods)^27^. The synthesized graphene was transferred onto the Al_2_O_3_ film by a poly(methyl methacrylate) (PMMA) assisted wet transfer method. The graphene was patterned by Al pre-patterning and subsequent O_2_ plasma etching. The lateral dimension of patterned graphene was 0.8 × 1.0 mm. On the transferred graphene, Al_2_O_3_ was deposited again through the ALD process. Finally, the top electrode (Cr/Au of 5 nm/70 nm) was deposited by thermal evaporation with a shadow mask for capacitance measurements. The chemical and structural characterization of Al_2_O_3_ and the interface properties between graphene and Al_2_O_3_ were investigated using X-ray photoelectron spectroscopy (XPS), Raman spectroscopy, and transmission electron microscopy (TEM). As shown in the cross-sectional TEM image (Fig. 1b), the thickness of Al_2_O_3_ films on ITO and graphene was about 40 nm, and a graphene sheet inserted in Al_2_O_3_ also was observed. Fig. 1c reveals Raman spectra of the single, bi-, and tri-layer graphene transferred onto Al_2_O_3_, clearly showing the graphene fingerprints, i.e., D-, G-, and 2D-bands. Because the intensity of the D-band was extremely small, the graphene was nearly defect-free and has a large domain size. We also confirmed the number of graphene layers through the ratio of 2D and G peaks (Supplementary Fig. S1a, b). After the formation of the Al_2_O_3_ layer onto graphene by ALD, highly-crystalline graphene was well-preserved, as confirmed by the intensity of the D-band (Supplementary Fig. S1c). The chemical identification of Al_2_O_3_ and graphene was carried out using XPS analysis. Figure 1d shows the C 1s core level spectra obtained from graphene on Al_2_O_3_, indicating that sp^2^ C-C bonds, a small C-O bond, and C = O bond were observed. Figure 1e exhibits a peak at 74 eV, corresponding to the Al 2p of Al_2_O_3_. These results reveal that the graphene and Al_2_O_3_ layers are well-formed. It is well known that the deposition of few nm-thick Al_2_O_3_ layers onto graphene by ALD is difficult because of the inert and hydrophobic surface of graphene. In our study, however, the 40 nm-thick Al_2_O_3_ layer was utilized for the formation of the Al_2_O_3_-graphene hybrid layer. In the formation of the Al_2_O_3_ layer on graphene, dangling bonds or functional groups on the graphene sheet can react with ALD precursors on the edges and defect sites, and Al_2_O_3_ layer was deposited uniformly by using these defects as staring nucleation sites. The uniformity of the top Al_2_O_3_ layer on graphene was examined by atomic force microscopy (AFM). The RMS roughness was about 0.305 nm, which was compared with Al_2_O_3_ on ITO (Supplementary Fig. S2).

### Dielectric properties of a graphene-embedded Al_2_O_3_ capacitor

**Figure 2:**
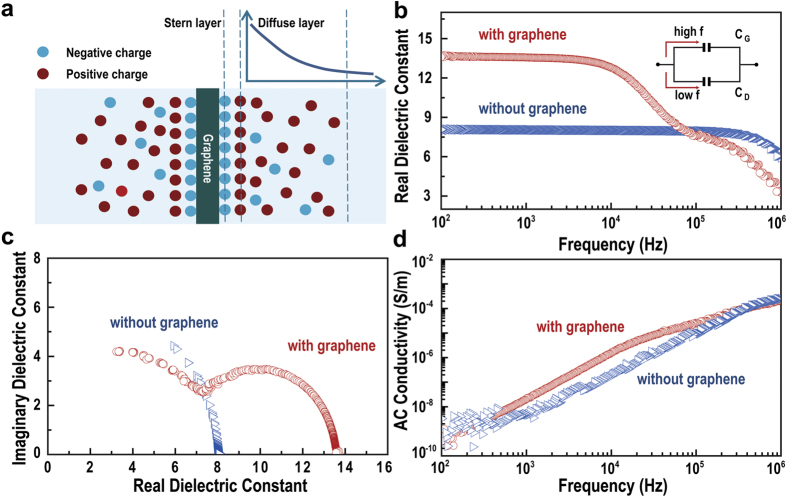
Dielectric properties of a graphene-embedded Al_2_O_3_ capacitor. (**a**) Schematic diagram to describe the formation of EDL at the graphene/Al_2_O_3_ interface. (**b**) Real dielectric constant as a function of frequency for the Al_2_O_3_ capacitor and graphene-embedded capacitor, inset is the equivalent circuit at different frequency ranges. (**c**) Nyquist curves, (**d**) ac conductivity for the Al_2_O_3_ and graphene-embedded capacitors.

Figure 2a shows a schematic diagram describing the formation of a double layer in Al_2_O_3_/graphene/Al_2_O_3_, where the induced potential from space charge polarization is plotted in terms of the position in the diffuse layer. Negative and the positive charges are accumulated on the surface of graphene and Al_2_O_3_, respectively. The local internal fields are enhanced by space charge polarization, so a set of EDLs is formed at the interface. Thus, the total capacitance is governed by these EDLs, and is the main factor increasing the dielectric constant. In Fig. 2b, the dielectric constants of Al_2_O_3_ capacitors with/without graphene sheets are plotted as a function of frequency. For the Al_2_O_3_ capacitor without graphene, the dielectric constant is about 8 at a frequency below 500 kHz, which remains constant down to 100 Hz. On the other hand, when graphene is inserted, two discernible dielectric constants are observed: about 8 at frequencies above 100 kHz and about 13.5 at frequencies below 10 kHz. This phenomenon can be explained by the formation of an EDL due to space charge polarization, as described above. The inset in Fig. 2b is an equivalent circuit that represents the dominant capacitance at different ranges of applied frequency. In the higher frequency region, the total capacitance is affected by the geometric capacitance. In the lower frequency region, the total capacitance is enhanced significantly due to the effect of EDL capacitance. This frequency dependence of the capacitance response can be explained by the time constant difference between the dielectric material and the EDL interface. Figure 2c shows the real and imaginary dielectric constants of the Al_2_O_3_ capacitor and graphene-embedded Al_2_O_3_ capacitor by plotting a Nyquist curve. Interestingly, unlike the Nyquist curve of the Al_2_O_3_ capacitor, the curve of the graphene-embedded capacitor consists of two semicircles, those which represent the relaxation behavior of the dipole polarization of Al_2_O_3_ (left semicircle) and the space charge polarization (right semicircle) at the interface. The relaxation time, τ_0_, equals the reciprocal of the frequency at the local maxima of the semicircles; hence, the calculated relaxation time of space charge polarization is about 5.78 μs, which means that the response time is appropriate for practical applications. The correlation between ac-conductivities and frequency for both capacitors is plotted in Fig. 2d, indicating that the ac-conductivity decreases with decreasing frequency. When conductivity is measured at even lower frequencies, we expect to see a plateau where value equals the conductance from leakage. Considering that ac-conductivity already is as low as 1.0 × 10^−10^ S/m at 100 Hz, we can safely assume that actual leakage is even lower and conclude that leakage is negligible.

### Formation of EDL at graphene/Al_2_O_3_ interface

**Figure 3:**
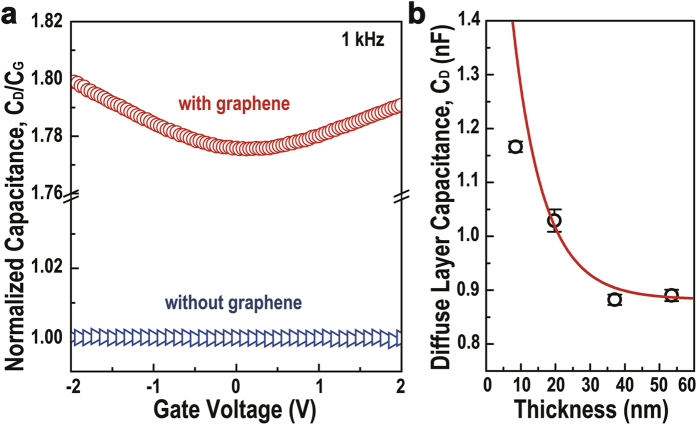
Gate potential dependence of capacitance. (**a**) Normalized capacitance as a function of gate voltage at 1 kHz frequency for the Al_2_O_3_ and graphene-embedded capacitors. (**b**) Diffuse layer capacitance as a function of upper Al_2_O_3_ thickness and fitting plot from the Gouy-Chapman EDL model (red line).

In order to analyze the interface in more detail, we measured the change in capacitance with respect to applied gate potential (Fig. 3a). As depicted in Fig. 2a, a set of EDLs consists of a Stern layer and a diffuse layer lined up in a series arrangement. Hence, the apparent capacitance should be limited by the layer that has lower capacitance than the others. Since, for an insulating material such as Al_2_O_3_, charge carrier concentration is rather diffuse, the capacitance from the diffuse layer is expected to be limiting. Figure 3a shows the normalized capacitance as a function of gate voltage at a frequency of 1 kHz for an Al_2_O_3_ capacitor and a graphene-embedded capacitor. A frequency of 1 kHz was adopted for this measurement because we expect to see the EDL behavior only at a frequency lower than 10 kHz. We observed gate voltage-independent behavior in capacitance for the Al_2_O_3_ capacitor without graphene, which is similar to the graphene-embedded capacitor at frequencies of 100 kHz and higher (Supplementary Fig. S3). However, the capacitance of the graphene-embedded capacitor is strongly dependent on gate potential at frequencies lower than 10 kHz. These results clearly suggest that improvement of capacitance occurred due to the formation of the diffuse layer at the interface between graphene and Al_2_O_3_. The amount of charges at the interface increases with increasing the gate potential, and the diffuse layer capacitance also increases. The effect of the potential of diffuse layer capacitance in the EDL can be expressed by the Gouy-Chapman model, which is given by^28^


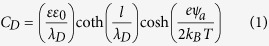


where ε_o_ is the permittivity in vacuum, *ε* is the relative dielectric constant of the material, λ_D_ is the width of diffuse layer (or Debye length), *l* is the geometrical thickness, 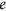 is the electron charge, 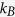 is the Boltzmann constant, and ψ_*a*_ is the applied potential. The total capacitance of the graphene-embedded capacitor consists of two diffuse layer capacitances in series because two interfaces are formed: one between the upper Al_2_O_3_ layer and graphene, and another between graphene and the lower Al_2_O_3_ layer. Thus, total capacitance in the low frequency region is given by:


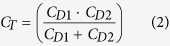


where *C*_*D*1_ and *C*_*D*2_ are the diffuse layer capacitances of the upper and the lower layer, respectively. The total capacitance was slightly asymmetric with the direction of dc bias, causing a slight shift in the C–V curve. There are two possible reasons for this; one is that the formation of a direction-dependent potential due to the work function difference between the upper (Au) and lower (ITO) electrodes. Another is that the difference in dielectric constants of upper and lower Al_2_O_3_ layers is presumably due to the difference in surface conditions when depositing the layers. To extract the value of 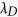 and dielectric constants of each layer, the minimum capacitance obtained by C–V measurements was plotted with various thicknesses (8.5, 20, 37.1, and 53.5 nm) of the upper Al_2_O_3_ layer. The result was fitted by combining Equations (1 and 2) (Fig. 3b). The values of 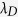 for both upper and lower Al_2_O_3_ layers by fitting are approximately 19 and 27 nm, and dielectric constants are 4 and 8, respectively. The dielectric constant of upper Al_2_O_3_ layer is lower than that of the lower layer^29^,^30^. When the thickness of the upper Al_2_O_3_ layer decreases below the calculated 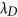 (19 nm), the experimental result could not be fitted very accurately, because the diffuse layer model is made for a semi-infinite system; hence, the case where the sample thickness is smaller than the 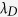 is inappropriate for this model.

**Figure 4:**
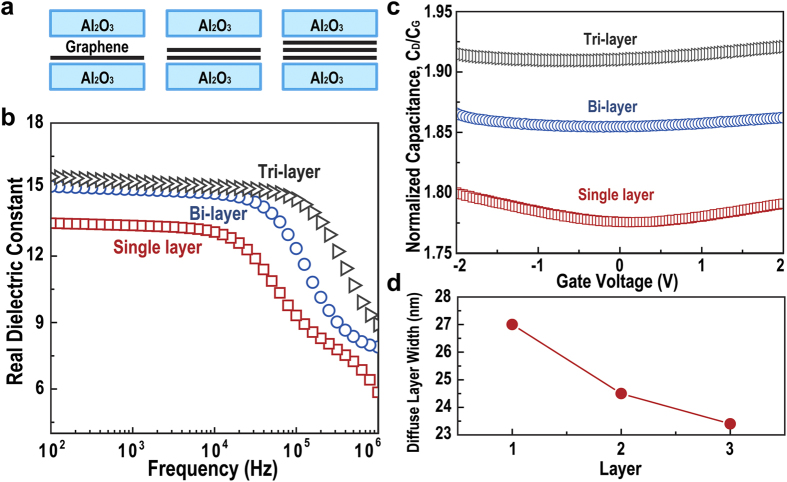
Diffuse layer capacitance and width for the number of graphene layers 1–3. (**a**) Schematic of Al_2_O_3_ capacitor with various numbers of graphene layers (1, 2, and 3). (**b**) Real dielectric constant as a function of frequency. (**c**) Normalized capacitance dependence on gate voltage, (**d**) calculated width of the diffuse layer for the number of graphene layers.

Single, bi-, and tri-layer graphene sheets were inserted in Al_2_O_3_ to explore the effects of the number of graphene layers to the diffuse layer formed at the interface, where a PMMA layer-by-layer transfer technique^31^ was used, as depicted in Fig. 4a. Figure 4b shows the plot of the dielectric constant vs. frequency for single, bi-, and tri-layer graphene-embedded capacitors. The dielectric constant increases slightly from 13.5 (for single layer graphene) to 15.5 (for tri-layer graphene), and the relaxation time of space charge polarization decreases from 5.78 to 0.36 μs with an increasing number of layers, as calculated from the dielectric loss spectrum (Supplementary Fig. S4), indicating that tri-layer graphene exhibits higher performance than single and bi-layer graphene-embedded capacitors as a gate dielectric. The improved dielectric performance with tri-layer graphene can be explained by the charge transfer at the graphene/Al_2_O_3_ interface. It was reported in previous literature that the hole concentration in graphene is proportional to the number of graphene layers^32^. Negative charges near graphene are well accumulated due to electrostatic force; hence, the carrier concentration in the diffuse layer increases, and the diffuse layer becomes narrow. However, over few layers of graphene, the carrier concentration in graphene would be saturated. Then, the dielectric constant also would no longer increase. The variation in carrier concentration can be confirmed by the curvature of C–V plots (Fig. 4c). According to the Gouy-Chapman EDL model, if the EDL is less diffuse, the total capacitance is decided by the Stern layer capacitance. In this case, the capacitance is independent of gate potential. However, the dependence of capacitance on applied voltage for all single, bi-, and tri-layer graphene-embedded capacitors was observed in C–V measurements (Fig. 4c). The curvature of C–V plots decreases with increasing number of graphene layers, indicating that the carrier concentration in the diffuse layer of a tri-layer capacitor is higher than that of a single and bi-layer capacitor. The diffuse layer narrows, and its capacitance also increases with increasing number of graphene layers. The values of λ_D_ calculated from Equations (1 and 2) decrease from 27 to 23.4 nm as the number of graphene layers increases, as seen in Fig. 4d.

## Discussion

We attribute the formation of EDL in graphene-embedded Al_2_O_3_ to charge carrier transfer at the graphene/Al_2_O_3_ interface. The charge carrier transport will induce a highly intense accumulation of negative charges on graphene (inner Helmholtz layer); then, the positive charges form a diffuse layer at the interface between the graphene and Al_2_O_3_ due to the Coulombic attraction, forming the EDLs as stated above. Since Al_2_O_3_ is an insulator with low carrier concentration, the diffuse layer is typically broad and becomes the limiting factor in induced capacitance. When the graphene layer is increased from a single to a tri-layer, the diffuse layer becomes narrow, indicating that charge carrier concentration within the diffuse layer has increased. Such an increase in charge carrier concentration occurs because electrostatic interaction has become stronger at the graphene/Al_2_O_3_ interface due to increasing carrier density within graphene caused by band structure overlap.

In summary, we proposed a graphene-embedded Al_2_O_3_ capacitor with a relatively high dielectric constant of 15.5 and low leakage current, especially with insertion of tri-layer graphene. For characterization of the interface between graphene and Al_2_O_3_, we measured the frequency and gate voltage dependence of capacitance by varying the thickness of the Al_2_O_3_ layer and varying the number of graphene layers for further analysis of the space charge layers formed on both sides of the graphene. Results obtained from various thicknesses of Al_2_O_3_ indicate that such enhancement of capacitance occurs due to the formation of a pair of EDLs at the graphene/Al_2_O_3_ interface. Capacitance dependence on applied gate voltage measurements suggest that the diffuse layer of EDL is the limiting and controlling factor of the capacitance enhancement, which is reasonable since Al_2_O_3_ can be considered as a very diffuse system with low carrier concentration. We also calculated the width of the diffuse layer for single, bi-, and tri-layer graphene-embedded capacitors, and they were 27.0, 24.5, and 23.3 nm, respectively. This result was explained by the change of carrier density in graphene. Even though the proposed dielectric material is not suitable for high-frequency applications such as radio frequency devices due to the limitation of response time, the enhancement of capacitance for a gate dielectric material with such a simple fabrication method can be considered a valuable asset in rapid-free device applications such as sensors and integrated circuit for display. Other properties of graphene, such as its superior mechanical strength, can make this method applicable in devices for various purposes, making graphene-insulator heterostructures even more appealing.

## Methods

### CVD growth of grapheme

Graphene was synthesized on 25 μm-thick copper (Cu) foils (Sigma Aldrich) using conventional TCVD. Cu foils were located in the quartz tube and pre-annealed under H_2_ (2 Torr) atmosphere at 1050 °C for 30 min, after which 1:50 mixture of CH_4_ and H_2_ was introduced for 20 min. The samples were cooled down to room temperature to complete the synthesis.

### ALD growth of Al_2_O_3_

For the deposition of Al_2_O_3_, trimethylaluminum (TMA) was employed as the precursor with H_2_O reactant as the oxidant. Each cycle consists of the TMA exposure of 0.3 s, Ar purging of 10 s, water vapor exposure of 1 s, and another Ar purging of 10 s in sequence at the substrate temperature of 100 °C. Total of 300 cycles was performed.

### Measurement of dielectric properties

All room temperature dielectric measurements were performed using an Agilent 4284A impedance analyzer. The measurement of dielectric performance of the Al_2_O_3_ capacitors with/without graphene sheet was conducted over the frequency range of 100 Hz–1 MHz with ac amplitude of 50 mV.

## Additional Information

**How to cite this article**: Ki Min, B. *et al.* Electrical Double Layer Capacitance in a Graphene-embedded Al_2_O_3_ Gate Dielectric. *Sci. Rep.* **5**, 16001; doi: 10.1038/srep16001 (2015).

## Supplementary Material

Supporting Information
