# Supplementary material for: Electrical Double Layer Capacitance in a Graphene-embedded Al2O3 Gate Dielectric
Source: Sci Rep. 2015 Nov 4;5:16001. doi: 10.1038/srep16001 (PMC4632157; doi:10.1038/srep16001)
Supplement: Supporting Information [file srep16001-s1.doc]

Supplementary Information:

**Electrical Double Layer Capacitance in a Graphene-embedded Al_2_O_3_ Gate Dielectric**

Bok Ki Min^1,2^, Seong K. Kim^3^, Seong Jun Kim^1^, Sung Ho Kim^1^, Min-A Kang^1^, Chong-Yun Park^2^, Wooseok Song^1^, Sung Myung^1^, Jongsun Lim^1^, and Ki-Seok An^1*^

^1^Thin Film Materials Research Group, Korea Research Institute of Chemical Technology (KRICT), Yuseong P. O. Box 107, Daejeon 305-600, Republic of Korea.

^2^Department of Physics, Sungkyunkwan University, 2066 Seobu-ro, Jangan-gu, Suwon-si, Gyeonggi-do 440-746, Republic of Korea.

^3^Department of Chemical Engineering and Materials Science, University of California, Davis, California 95616-5294, USA.

Correspondence and requests for materials should be addressed to K.-S. An (email: ksan@krict.re.kr).


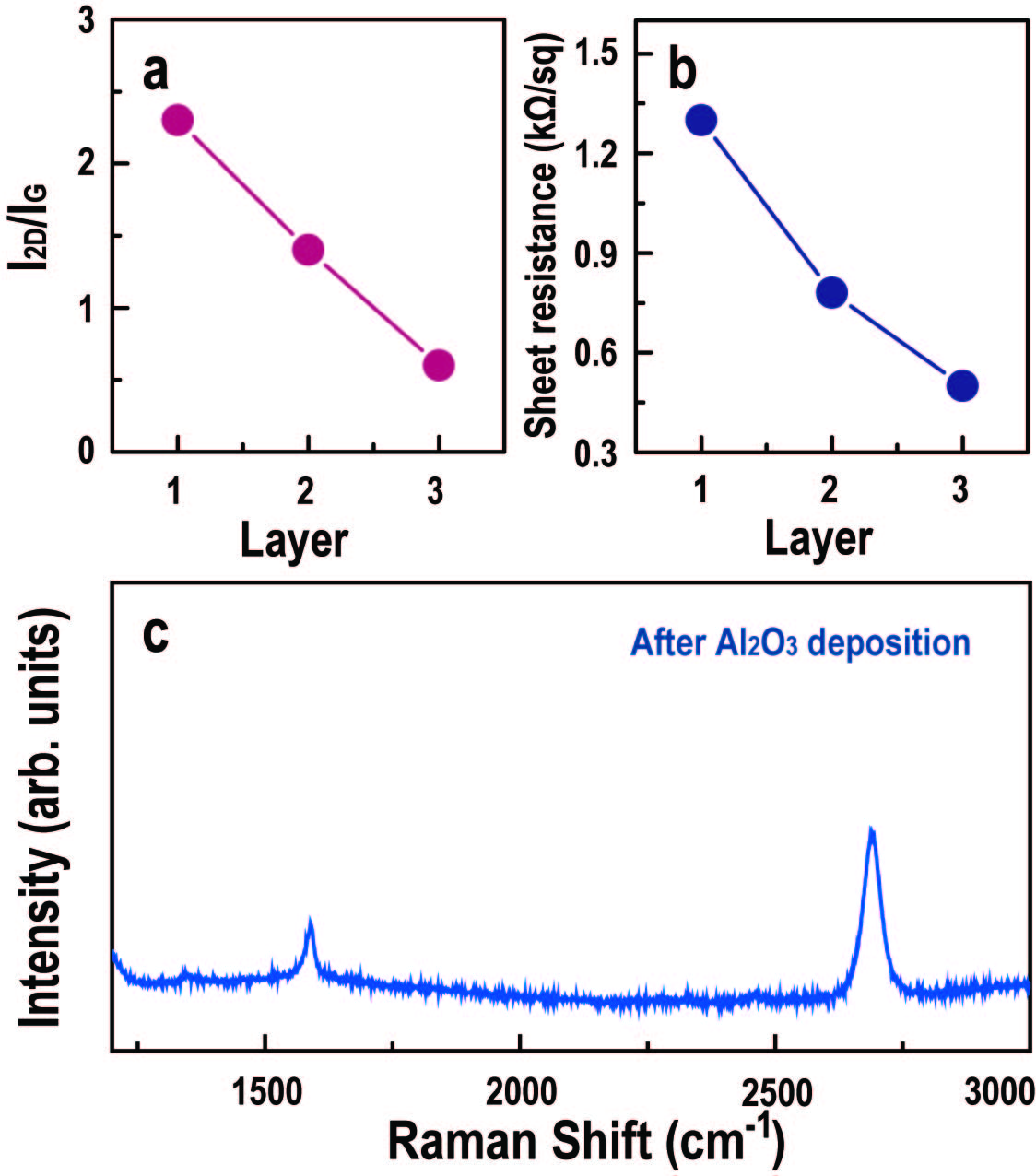


**Figure S1.** (a) Intensity ratio of 2D and G peak position, (b) Sheet resistance measured by 4-point probe equipment as the number of graphene layers. (c) Raman spectra of graphene after deposition of Al_2_O_3_ layer.


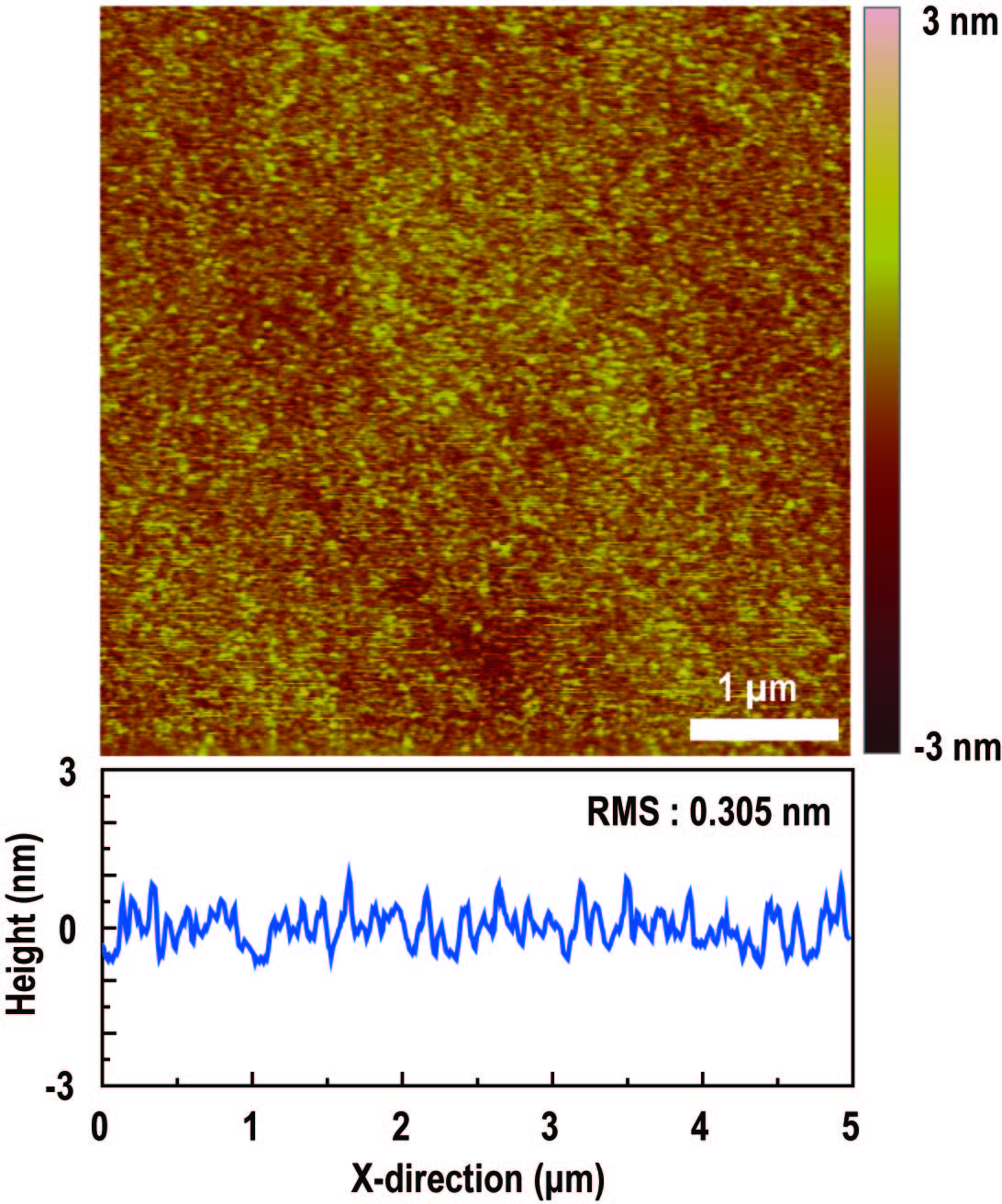


**Figure S2.** AFM image and height profile of Al_2_O_3_ film deposited by ALD on graphene.


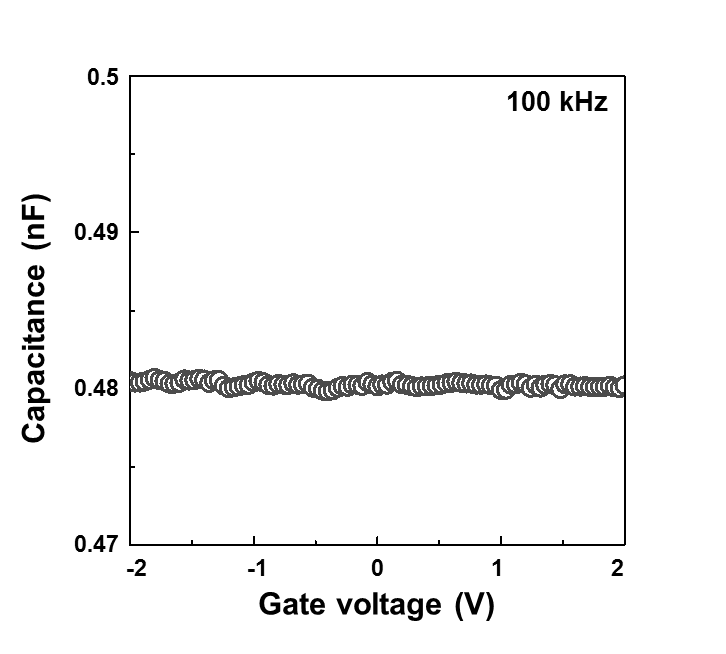


**Figure S3.** Capacitance as a function of gate voltage at 100 kHz of frequency for Al_2_O_3_ capacitor with mono-layer graphene.


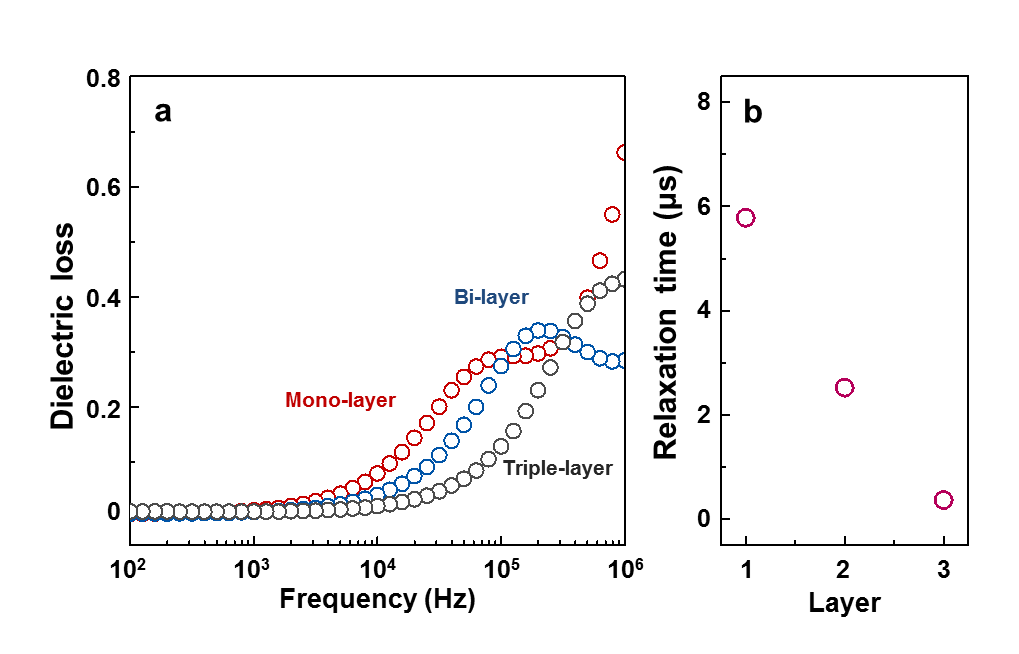


**Figure S4.** (a) Frequency dependence of dielectric loss tangent of Al_2_O_3_ capacitor with different layers of graphene. (b) Relaxation time of space charges as the number of graphene layers.
